# Development of a Flow Cytometry Assay to Predict Immune Checkpoint Blockade-Related Complications

**DOI:** 10.3389/fimmu.2021.765644

**Published:** 2021-11-16

**Authors:** Hannah-Lou Schilling, Gunther Glehr, Michael Kapinsky, Norbert Ahrens, Paloma Riquelme, Laura Cordero, Florian Bitterer, Hans J. Schlitt, Edward K. Geissler, Sebastian Haferkamp, James A. Hutchinson, Katharina Kronenberg

**Affiliations:** ^1^ Department of Surgery, University Hospital Regensburg, Regensburg, Germany; ^2^ Institute of Functional Genomics and Statistical Bioinformatics, University of Regensburg, Regensburg, Germany; ^3^ Beckman Coulter Germany, Krefeld, Germany; ^4^ Medizinisches Versorgungszentrum (MVZ) for Laboratory Medicine Raubling, amedes Labor, Raubling, Germany; ^5^ Institute of Clinical Chemistry and Laboratory Medicine, University Hospital Regensburg, Regensburg, Germany; ^6^ Department of Dermatology, University Hospital Regensburg, Regensburg, Germany

**Keywords:** flow cytometry, assay validation, immune checkpoint inhibition, immune-related adverse events, prediction, effector memory T cells, monocytes, biomarker

## Abstract

Treatment of advanced melanoma with combined immune checkpoint inhibitor (ICI) therapy is complicated in up to 50% of cases by immune-related adverse events (irAE) that commonly include hepatitis, colitis and skin reactions. We previously reported that pre-therapy expansion of cytomegalovirus (CMV)-reactive CD4^+^ effector memory T cells (T_EM_) predicts ICI-related hepatitis in a subset of patients with Stage IV melanoma given αPD-1 and αCTLA-4. Here, we develop and validate a 10-color flow cytometry panel for reliably quantifying CD4^+^ T_EM_ cells and other biomarkers of irAE risk in peripheral blood samples. Compared to previous methods, our new panel performs equally well in measuring CD4^+^ T_EM_ cells (agreement = 98%) and is superior in resolving CD4^+^ CD197^+^ CD45RA^-^ central memory T cells (T_CM_) from CD4^+^ CD197^+^ CD45RA^+^ naive T cells (T_naive_). It also enables us to precisely quantify CD14^+^ monocytes (CV = 6.6%). Our new “monocyte and T cell” (MoT) assay predicts immune-related hepatitis with a positive predictive value (PPV) of 83% and negative predictive value (NPV) of 80%. Our essential improvements open the possibility of sharing our predictive methods with other clinical centers. Furthermore, condensing measurements of monocyte and memory T cell subsets into a single assay simplifies our workflows and facilitates computational analyses.

## Introduction

Immune checkpoint inhibitor treatment, particularly combined PD-1/CTLA-4 blockade, has dramatically improved treatment response and overall survival rates for patients with inoperable metastatic melanoma (1–6). Unfortunately, immune-related adverse reactions, such as hepatitis and colitis, are common complications that range in severity from mild reactions to life-threatening conditions (7–12). Methods to predict adverse events or tumor responses would have great clinical utility in guiding optimal therapy. Various predictive markers of adverse reactions have been reported in recent literature (13–25), including our own discovery that elevated CD4^+^ effector memory T cell (T_EM_) frequency in the blood is strongly associated with hepatitis risk after αPD-1 and αCTLA-4 treatment (26).

Our studies showed that exaggerated cytomegalovirus (CMV)-specific memory T cell responses cause liver injury in some patients with advanced melanoma receiving αPD-1 (Nivolumab) and αCTLA-4 (Ipilimumab) therapy. This surprising discovery stemmed from our observation that pre-treatment expansion of CD4^+^ effector memory T cells (T_EM_) in the blood reliably predicts hepatitis. We assume that chronic or recurrent CMV reactivation drives expansion of virus-specific CD4^+^ T_EM_ cells long before starting immunotherapy (27–30); however, we are presently unable to explain why patients with metastatic melanoma might be especially susceptible to subclinical CMV reactivation. Understanding the mechanistic basis of our predictive model was important because it led us to realise that ICI-related hepatitis could be prevented with prophylactic valganciclovir. Promisingly, 4 of 4 patients classified as high-risk for hepatitis who received CMV prophylaxis starting from their first exposure to αPD-1/αCTLA-4 did not develop hepatitis (26).

Notably, our proposed etiology does not account for hepatitis in CMV-uninfected individuals, which necessarily implies that checkpoint blockade-related hepatitis has more than one cause. Hepatitis in $\text{CD}4^{+}{\text{T}}_{\text{E}}^{\text{M}}$ ^low^ patients tends to occur in younger individuals with lower numbers of circulating CD14^+^ monocytes.

We plan to conduct a randomised, prospective, multicenter clinical trial to test the efficacy of valganciclovir prophylaxis in preventing hepatitis in CD4^+^ T_EM_^high^ patients. Impacting our plans, the European Union passed new regulations about *in vitro* diagnostics (IVD) into law in 2017 (31). After a 5-year transition period, this Regulation will be mandatory from 26^th^ May, 2022. The IVD Regulations (IVDR) set high quality and security standards. Consequently, licensing of IVD assays in Europe will require manufacturers to demonstrate the scientific validity, analytical performance and clinical performance of their product for a given indication. The scientific validity of measuring CD4^+^ T_EM_ % as a risk-predictor for checkpoint blockade-related hepatitis was firmly established in our earlier studies (26). Our planned clinical trial should establish the clinical utility of our predictive models.

In this report, we describe the development of an optimised flow cytometry-based assay that consolidates measurement of CD4^+^ T_EM_ cells, CD3^+^ T cells, PD-1^+^ CD8^+^ T cells and CD14^+^ monocyte frequency into a single test. This technical development streamlines sample handling in our daily clinical routine by reducing sample processing time and minimizes opportunities for technical error. In addition to assessing the analytical performance of this new assay, we investigated patient-related factors that might influence its proper interpretation. The robust preclinical performance of our “monocyte and T cell” (MoT) assay justifies its adoption for future multicentre clinical trials.

## Results

### Panel Design and Optimization

**Figure 1:**
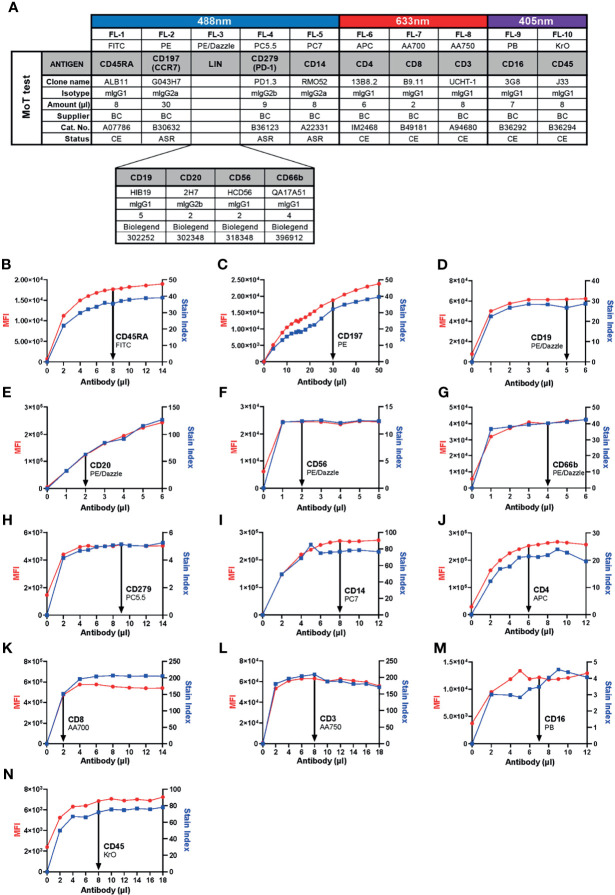
Development of the 10-color “MoT test” to concurrently analyze CD3^+^ T cells, CD4^+^ T_EM_ cells, CD279^+^ CD8^+^ T cells and CD14^+^ monocytes. **(A)** Antibodies against CD45, CD3, CD4 and CD8 were used as T cell backbone. Lineage antibodies against CD19, CD20, CD56 and CD66b were used for exclusion of B cells, NK cells and granulocytes. Antibodies against CD45RA and CCR7 were used for the characterization of memory T cell subsets. αCD279 was used to characterize late-activated and exhausted T cells. αCD14 and αCD16 were used for subsetting monocytes. **(B–N)** Titration of antibodies included in the MoT test. Optimal concentrations were chosen according to MFI and Stain Index.

Previous work identified a high CD4^+^ T_EM_ cell frequency in blood prior to therapy as a risk marker for PD-1/CTLA-4-related hepatitis in patients with advanced melanoma (26). In those studies, CD4^+^ T_EM_ cells were defined as CD45^+^ CD3^+^ CD4^+^ CD8^-^ CD45RA^-^ CD197^-^ events. With a view to continuity of our assay principle, this definition was not changed in our new assay. Likewise, we kept the same definition of monocytes from our previous studies – namely, CD45^+^ CD14^+^ mononuclear cells. We included CD16 into our new panel to allow a more refined subsetting of classical, intermediate and non-classical monocytes. Other groups have identified CD279 (PD-1) expression in CD8^+^ T cells as a marker of clinical response after αPD-1/αCTLA-4 treatment in patients with melanoma (32–37); therefore, our panel also enables quantification of CD45^+^ CD3^+^ CD4^-^ CD8^+^ CD279^+^ events. To improve the accuracy of our cell type definitions, we included a lineage exclusion (Lin) channel to gate-out CD66b^+^, CD56^+^, CD19^+^ or CD20^+^ events. Because this panel is intended for use with fresh whole blood samples, discrimination between dead and live cells was unnecessary. Hence, our new panel included 10 parameters: CD45, Lin, CD3, CD4, CD8, CD45RA, CD197 (CCR7), CD279 (PD-1), CD14 and CD16 (**Figure 1A**).

Because we intended to develop our new panel as an IVD assay, we prioritized selection of reagents with CE/IVD labels from manufacturers with robust supply chains. With the intention of preserving as much of the original assay design as possible, T cell markers were assigned to the same channels and fluorochromes used by the DURAClone IM T cell Subset kit. Identical monoclonal antibodies (mAb) were selected for CD45, CD3, CD4, CD8, CD197 and CD279. When we compared two alternative FITC-conjugated mAb clones, we found ALB11 (Stain Index = 30.0) resolved CD45RA expression better than 2H4 (Stain Index = 14.6; **Supplementary Figure 1**). For this reason, and considering ALB11 was supplied as a CE/IVD grade reagent, we substituted 2H4-FITC with ALB11-FITC in the revised panel that was used for this study.

In previous work, we detected monocytes with CD14-PE-Cy7 (clone RMO52) and CD16-FITC (clone 3G8). For continuity, we kept CD14-PE-Cy7; however, to accommodate our T cell markers, CD16-FITC was switched for CD16-PB. Our selection of antibodies left only PE/Dazzle open for lineage exclusion.

Six of thirteen selected mAbs carry CE/IVD marking, indicating compliance with requirements for IVD products pursuant to the European Union (EU) Directive 98/79/EC. Three other mAbs are labelled as *Analyte Specific Reagents* (ASR), which certifies them as suitable reagents for use as active ingredients in IVD kits. All four Lin mAbs meet ISO 13485 standards. Hence, from a regulatory perspective, all antibodies chosen for our redesigned panel are suitable for inclusion into a clinical assay (38).

Next, we determined optimal concentrations of each antibody to obtain the best resolution of stained cell populations. Each antibody was titrated against fresh whole EDTA blood from healthy donors to determine a saturating amount of antibody that optimized Stain Index (**Figures 1B–N**).

### Gating Strategy and Reported Parameters

**Figure 2:**
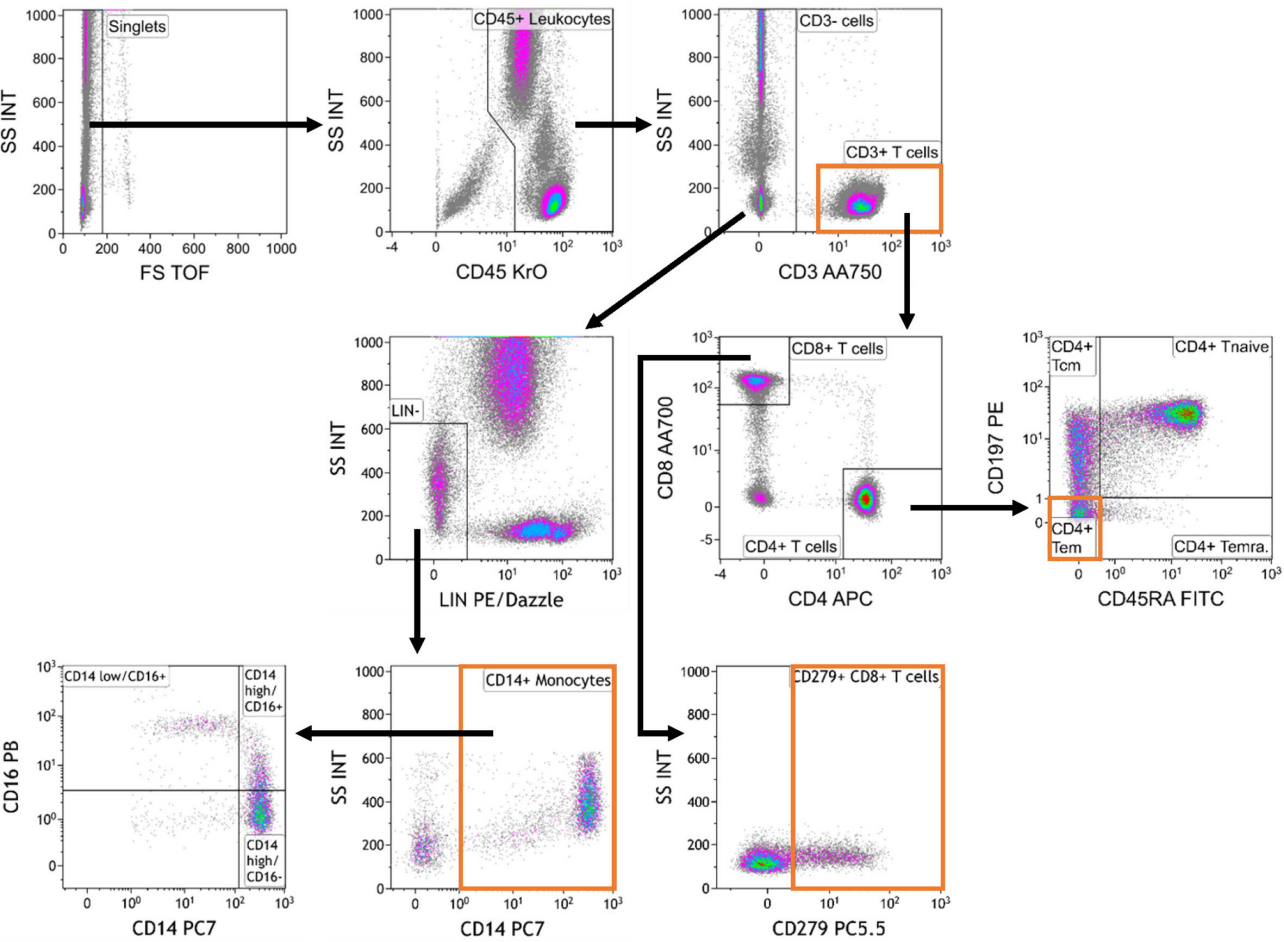
Gating strategy used for analysis of the MoT test.. Data were analyzed as follows: Selection of singlets; gating on CD45^+^ leukocytes; gating on CD3^+^ T cells and CD3^-^ cells; gating on Lineage-negative (Lin^-^) cells within the CD3^-^ compartment; gating on CD14^+^ monocytes; gating on monocyte subpopulations defined by CD14 and CD16 expression; gating on CD4^+^ and CD8^+^ T cells within the CD3^+^ T cell compartment; gating on CD4^+^ memory T cell subsets; gating on CD279^+^ CD8^+^ T cells.

Cell populations were identified according to a standardized gating strategy (**Figure 2**). Specifically, we report the frequency of (1) CD3^+^ T cells % with respect to CD45^+^ leucocytes, (2) CD4^+^ T_EM_ cell % with respect to CD4^+^ T cells, (3) CD14^+^ monocyte % with respect to CD45^+^ leucocytes and (4) CD279^+^ CD8^+^ T cell % with respect to CD8^+^ T cells. Our redesigned assay, which encompasses our choices about markers, antibodies, technical conditions, reported parameters and analysis strategy, was designated as the “MoT test”.

### Assay Performance Characteristics

#### Precision

The precision of an analytical method is an estimate of the agreement between measured results from one sample when the assay is performed repeatedly (39). Estimating intra- and inter-assay precision allows us to guarantee the reproducibility of the measured work within a defined range.

##### Intra-Assay Precision

**Figure 3:**
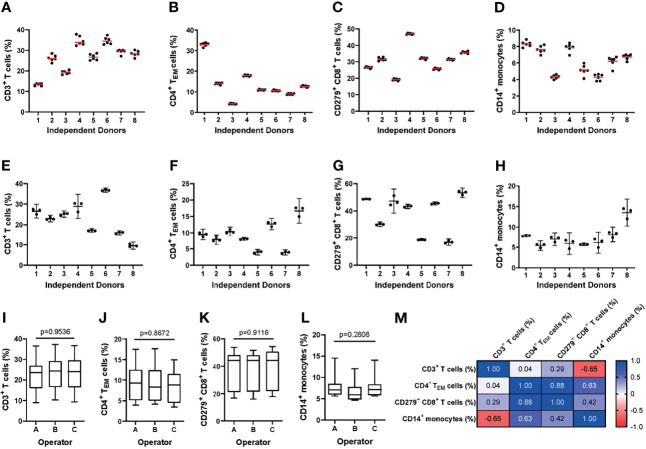
Evaluating the intra- and inter-assay precision of the MoT test. **(A–D)** For evaluation of intra-assay precision, 6 replicates from fresh whole blood EDTA samples from n = 8 healthy donors were stained using the MoT test. **(E–I)** For evaluation of inter-assay precision, fresh whole EDTA blood from n = 8 healthy donors was stained in parallel by three different operators. Frequencies of CD3^+^ T cells **(A, E)**, CD4^+^ T_EM_ cells **(B, F)**, CD279^+^ CD8^+^ T cells **(C, G)** and CD14^+^ monocytes **(D, H)** were determined (**A–D**: replicates are represented by dots, mean is displayed as red line; **(E–H)**: operator-dependent results are displayed as scatter dot plot with line at mean and 95% CI). **(I–L)** Reporter frequencies of inter-assay precision analyses (K.W. test). **(M)** Correlations (Pearson r) between reporters measured by the MoT test.

To assess intra-assay precision, we performed the MoT test with six replicates drawn from a single blood sample. One operator repeated this procedure with samples from 8 blood donors (**Figures 3A–D**). For all 4 reported values, the intra-assay precision mean CV was < 10% which is an acceptable level for such an assay (40) (**Supplementary Figure 2**).

##### Inter-Assay Precision

To assess inter-assay precision, three technicians each performed the MoT test with samples from 8 healthy blood donors in parallel (**Figures 3E–H**). The inter-assay precision mean CV for all 4 reporters was < 10% which is an acceptable level for such an assay (40) (**Supplementary Figure 3**). No statistically significant differences could be observed between operators for any of the reported values analyzed (**Figures 3I–L**). Correlations were observed between CD4^+^ T_EM_ cell % and CD8^+^ CD279^+^ T cell %, between CD4^+^ T_EM_ cell % and CD14^+^ monocyte %, as well as between CD3^+^ T cell and CD14^+^ monocyte % (**Figure 3M**).

##### Inter-Operator Analytical Precision

**Figure 4:**
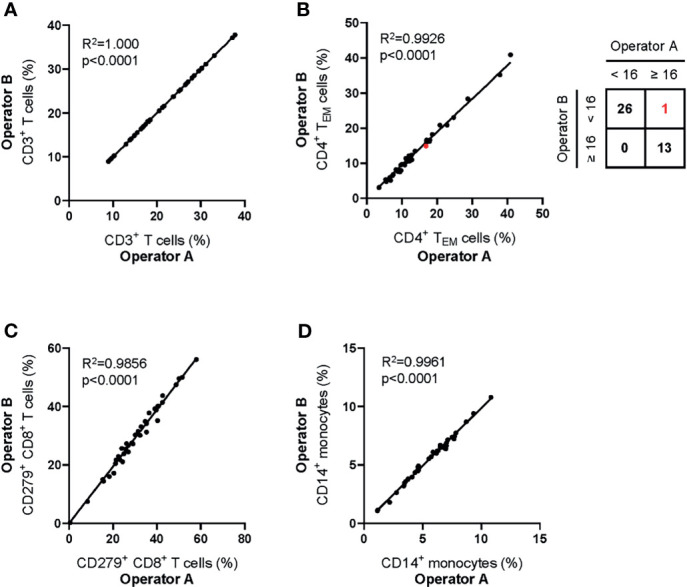
Inter-operator analytical precision of the MoT test. Fresh whole blood EDTA samples from n = 20 healthy donors and n=20 melanoma patients (stage II: n = 7; stage III: n = 3; stage IV: n = 10) were processed using the MoT test. Flow cytometry data were analyzed by two experienced operators according to the gating strategy shown in **Figure 2**. Frequencies of CD3^+^ T cells **(A)**, CD4^+^ T_EM_ cells **(B)**, CD279^+^ CD8^+^ T cells **(C)** and CD14^+^ monocytes **(D)** were determined. One sample of borderline CD4^+^ T_EM_ cell frequency was differently classified by the two operators (marked in red; inter-operator concordance of 98%).

To assess the reliability of manual gating of reported populations, two experienced operators made independent analyses of 40 data files obtained from healthy donors and patients with Stage II, III and IV melanoma. Inter-operator agreement for all reported parameters was high (**Figures 4A–D**). The operators agreed in 39/40 (98%) of cases about the classification of patients’ hepatitis risk using the previously reported cut-off value of CD4^+^ T_EM_ ≥ 16% (26).

#### Robustness

Robustness describes the susceptibility of an assay to changes in experimental conditions, both pre- and post-sample preparation (41). The purpose of stability testing is to define a range of conditions for sample handling that do not perturb assay performance. The most relevant conditions to consider in this case are sample storage temperature and storage duration before and after processing.

##### Pre-Processing Stability

**Figure 5:**
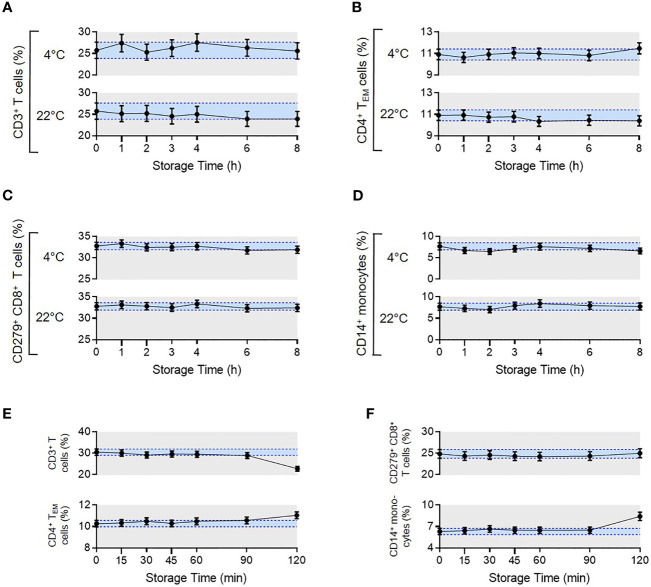
Pre- and post-analytical stability of the MoT test. To evaluate pre-analytical stability, whole blood EDTA samples from n = 4 healthy donors were either stored at 4°C in the dark in the fridge or light exposed at room temperature. Processing the samples started either directly after blood draw (0 h) or delayed in time (1-8 h). Acquired flow cytometry data were analyzed and frequencies of CD3^+^ T cells **(A)**, CD4^+^ T_EM_ cells **(B),** CD279^+^ CD8^+^ T cells **(C)** and CD14^+^ monocytes **(D)** were determined. Mean CVs of intra-assay precision were used for calculation of SDs. Results are displayed as mean with 95% CI. For the analysis of post-analytical stability, acquisition of fully processed blood samples from healthy donors (n = 3 for CD279^+^ CD8^+^ T cells, n = 6 for all other reporters) started either immediately after staining or delayed in time (15-120 min). **(E, F)** Frequencies of CD3^+^ T cells, CD4^+^ T_EM_ cells, CD279^+^ CD8^+^ T cells and CD14^+^ monocytes were determined. Mean CVs of intra-assay precision were used for calculation of SDs. Results are displayed as mean with 95% CI.

To assess pre-processing stability, blood samples from four healthy donors were either stored at 4°C or room temperature (RT). Sample processing was either started immediately (0h) or delayed until 1h, 2h, 3h, 4h, 6h and 8h after sample collection. No significant changes in reported parameters were observed over a storage time of 8h at 4°C or RT (**Figures 5A–D**).

##### Post-Processing Stability

To assess the stability of samples after preparation but prior to measurement, we compared blood samples from healthy donors that were processed immediately after blood draw. Measurements were then made directly after staining (0 min) or delayed until 15, 30, 45, 60, 90 or 120 min post-processing. Samples were stored at 4°C in the dark until data acquisition. Frequencies of all reported values were stable up to 90 mins (**Figures 5E, F**). Accordingly, data must be acquired within 90 mins of sample processing, which is feasible in our clinical routine.

#### Accuracy

Accuracy describes the closeness of agreement between the measured value and the true value. For most diagnostic tests, accuracy is assessed by calibrating measured values to some external reference material; however, as widely discussed in the literature (42–44), this is not an easy approach to apply to flow cytometry measurements.

##### External Reference Materials

In principle, the accuracy of a flow cytometry-based assay can be monitored over time against a standardized reference material; however, in practice, stabilizing cells for long-term storage often alters their optical qualities. We investigated whether two commercially available reference materials (i.e., Streck CD-Chex Plus and Beckman Coulter ClearLLab Control Cells Normal) were suitable controls for the MoT test. The physical properties, autofluorescence and staining intensity of cell-surface markers of cells from both preparations were not comparable to cells from fresh blood samples (**Supplementary Figure 4**). Therefore, these reference materials were unsuitable for our purposes.

##### Method Comparison

**Figure 6:**
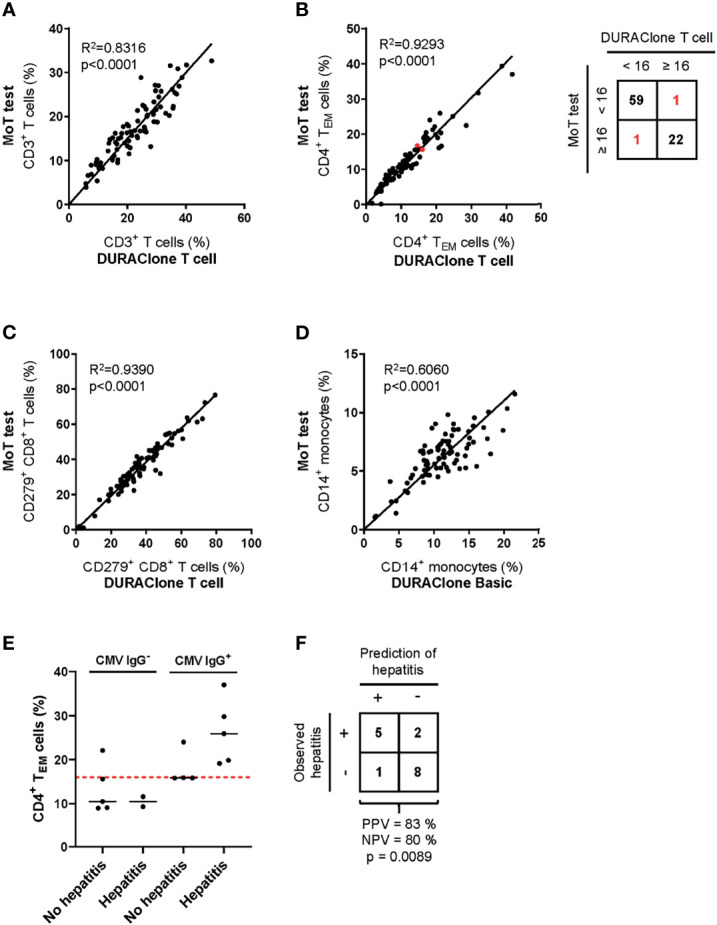
Method comparison of MoT test and DURAClone IM Tubes. Fresh whole blood EDTA samples from patients with stage II (n = 52), stage III (n = 11) or stage IV (n = 20) melanoma were investigated using the MoT test, the DURAClone IM Phenotyping Basic Tube and the DURAClone IM T cell subsets Tube. Frequencies of CD3^+^ T cells **(A)**, CD4^+^ T_EM_ cells **(B),** CD279^+^ CD8^+^ T cells **(C)** and CD14^+^ monocytes **(D)** were determined. The alternative methods differently classified only two samples with borderline CD4^+^ T_EM_ cell frequencies (marked in red; method concordance of 98%). Fresh whole EDTA blood of stage IV melanoma patients (n = 16) was analyzed prior to checkpoint inhibitor treatment using the MoT test. **(E)** CMV serology status was determined and the development of immune-related hepatitis was evaluated within 12 weeks post therapy start. Dashed red line indicates a cut-off of CD4^+^ T_EM_ ≥ 16%. Median values are indicated by a black line. **(F)** Classification of stage IV melanoma who did or did not develop immune-related hepatitis. Development of hepatitis was correctly predicted in 5 of 6 cases (PPV = 83%). 2 of 10 patients who developed hepatitis were incorrectly classified as hepatitis negative (NPV = 80%) (p = 0.0089, two-way ANOVA test).

To ensure continuity of our assay principle, we must be confident that we accurately quantify the same marker populations used to generate our predictive models (45). Therefore, we next compared results from previous panel designs to those generated with the MoT test. Samples from 83 melanoma patients with Stage II, III or IV disease were analyzed in parallel using the MoT test, DURAClone IM T cell subsets tube and DURAClone IM Phenotyping Basic tube (**Figures 6A–D**). A strong correlation between methods was observed for all reported parameters besides CD14^+^ monocytes, which showed a moderate correlation (R^2^ = 0.606). Our previous work established a cut-off ≥16% CD4^+^ T_EM_ cells as a predictor of checkpoint blockade-related hepatitis in CMV Ig^+^ patients (26). The DURAClone IM T cell subsets tube and MoT assay agreed in 81/83 cases (98%) when classifying patients as CD4^+^ T_EM_^≥16%^ or CD4^+^ T_EM_^<16%^ Furthermore, the MoT test performed well in predicting hepatitis in a cohort of 16 Stage IV melanoma patients (**Figure 6E**). Hepatitis was correctly predicted in 5 of 6 CMV IgG^+^ CD4^+^ T_EM_^≥16%^ melanoma patients, which corresponds to a PPV of 83% and NPV of 80% (**Figure 6F**).

### Age, Sex and Disease-Related Bias

**Figure 7:**
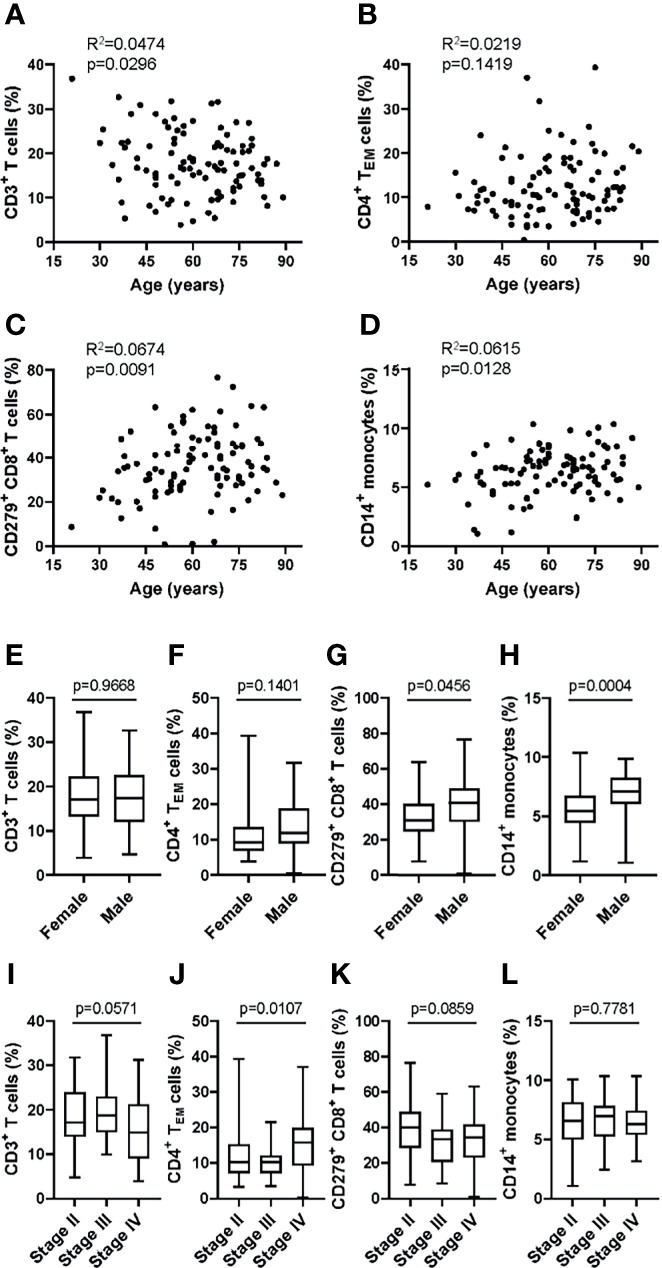
Application of the MoT test to a melanoma patient cohort revealed sex- and stage-dependent differences in T cell and monocyte populations. Fresh whole blood EDTA samples from patients with stage II (n = 52), stage III (n = 21) or stage IV (n = 27) melanoma were investigated using the MoT test. No correlation (Pearson) with age was found for CD3^+^ T cell % **(A),** CD4^+^ T_EM_ cell % **(B)**, CD279^+^ CD8^+^ T cell % **(C)** and CD14^+^ monocytes % **(D)**. No sex-dependent differences (unpaired t test) were found for CD3^+^ T cells **(E)** and CD4^+^ T_EM_ cells **(F)**. CD279^+^ CD8^+^ T cells **(G)** and CD14^+^ monocytes **(H)** were increased in male patients (unpaired t test). **(I)** CD3^+^ T cells were decreased in stage IV patients (K.W. test). **(J)** CD4^+^ T_EM_ cells showed a significant stage-dependent increase (K.W. test). No stage-dependent differences (K.W. test) were found in CD279^+^ CD8^+^ T cells **(K)** and CD14^+^ monocytes **(L)**.

To examine the applicability of the MoT test in a clinical setting, we analyzed blood samples from 100 patients with Stage II, III or IV melanoma. No relation to patient age was observed for CD3^+^ T cells (**Figure 7A**), CD4^+^ T_EM_ cells (**Figure 7B**), CD279^+^ CD8^+^ T cells (**Figure 7C**) or CD14^+^ monocytes (**Figure 7D**). Of interest, our analysis revealed higher frequencies of CD279^+^ CD8^+^ T cells (**Figure 7G**) and CD14^+^ monocytes (**Figure 7H**) in male patients. CD3^+^ T cells (**Figure 7E**) and CD4^+^ T_EM_ cells (**Figure 7F**) showed no sex-dependent differences. Notably, a tumor stage-dependent increase in CD4^+^ T_EM_ cells (**Figure 7J**) was observed. CD3^+^ T cells, CD279^+^ CD8^+^ T cells and CD14^+^ monocytes showed no stage-dependent differences (**Figures 7I, K, L**).

### Seasonality

Seasonality is an important environmental factor that influences immune responses (46, 47). Samples from 100 melanoma patients (Stage II, III and IV) which were stained using the MoT test were collected from December 2020 until June 2021. Season-dependent frequencies of immune cell populations were assessed (**Supplementary Figure 5**). CD4^+^ T cells showed a tendency to be increased in spring and summer whereas CD8^+^ T cells showed a tendency decrease.

### SPADE Analysis of Flow Cytometry Data Resulting From the MoT test

**Figure 8:**
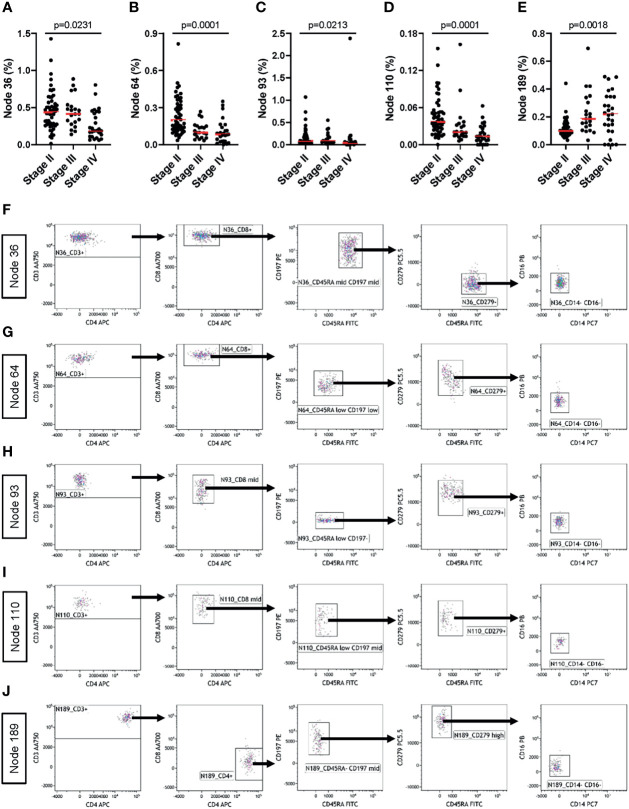
The MoT test captures information about tumor staging. Fresh whole EDTA blood from patients with stage II (n = 52), stage III (n = 21) or stage IV (n = 27) melanoma were investigated using the MoT test. SPADE analysis of Lin^-^ cells was performed in Cytobank with a density-dependent down-sampling target of 10% and a 200-cluster spanning tree. **(A–E)** Five clusters with significant stage-dependent differences were identified (K.W. test; B.H.-adjusted p-values, FDR = 5%). **(F–J)** All five significant SPADE clusters were re-exported and analyzed as follows: gating on CD3^+^ T cells; gating on CD8^+^ or CD4^+^ T cells; gating on memory T cell subsets (CD45RA/CD197); gating on CD279 cells and gating on CD14^-^/CD16^-^ cells.

To further explore tumor stage-related influences over the peripheral blood T cell and monocyte compartments, we analyzed data generated using the MoT test from patients with Stage-II (n=52), -III (n=21) or –IV (n=27) melanoma. Unsupervised grouping of flow cytometry data using SPADE defined 200 cell clusters (**Supplementary Figure 6**). Comparing cell frequencies in clusters representing >0.25% of all events (**Supplementary Figure 7**) revealed tumor stage-associated differences in five nodes (**Figures 8A–E**). Investigating these clusters in Kaluza (software version 2.1) revealed an association between tumor stage and CD279^-^ naïve CD8^+^ T cells (**Figure 8F**), CD279^+^ CD8^+^ T_CM_ (**Figure 8G**), CD279^+^ CD8^mid^ T_EM_ (**Figure 8H**) and CD279^+^ CD8^mid^ T_CM_ (**Figure 8I**) as well as CD279^high^ CD4^+^ T_CM_ (**Figure 8J**). Hence, apart from predicting the risk of immune-related adverse reactions in melanoma patients, the MoT test also captures some information about intra- and extra-nodal metastasis of melanoma.

## Discussion

Here, we developed and assessed the performance of a new flow cytometry-based assay for the prediction of checkpoint blockade-related complications. This assay is a refinement of methods used previously to develop a predictive model for hepatitis following combined αPD-1/αCTLA-4. Specifically, we adapted the assay design to include measurements of monocyte subsets together with CD279^+^ CD8^+^ T cells, which may be useful parameters for predicting treatment-related hepatitis, clinical response to therapy or tumor staging (48, 49).

The performance characteristics of the MoT test were satisfactory in all respects (50). Most importantly, we found good agreement between results from the MoT test and earlier methods; therefore, our developments have not changed the underlying principle of the assay. We now intend to have our new antibody panel custom-manufactured in a dried-down format, which is more convenient for routine diagnostic work and minimises technical variation (51, 52). Accordingly, the MoT test simplifies processing of samples, data collection and analysis without compromising accuracy, precision or robustness. These improvements will make it easier to establish our predictive method at collaborating clinical centres. Ultimately, we hope the MoT test will be registered as a CE/IVD device and implemented as a routine clinical method.

It is surprising that so much predictive information is reflected in the distribution of memory T cell and monocytes in blood of patients with melanoma. Combining all relevant parameters in a single dataset makes this information more amenable to algorithmic analysis. By collecting data from a very large number of patients, we expect to improve our prediction of checkpoint blockade-related complications and, perhaps, also to predict clinical outcomes or detect occult metastasis using a simple, relatively inexpensive test.

## Materials & Methods

### Study Approval and Patient Management

Peripheral blood samples were obtained from a heterogeneous group of healthy donors from the department of Transfusion Medicine at UKR or from Stage-II, -III, or –IV melanoma patients who participated in an observational clinical trial authorized by the local ethics committee (approval 16-101-0125). All participants gave full, informed written consent.

### Clinical Flow Cytometry

Detailed step-by-step protocols for preparation and analysis of blood samples by flow cytometry are available as **Supplementary Methods**. In brief, peripheral blood samples were collected into EDTA-vacutainers by venepuncture and then delivered to the immune monitoring laboratory at ambient temperature. Pre-analytical samples were stored for up to 4 hours at 4°C until processing. Whole blood was stained with DURAClone IM Phenotyping Basic Tube (B53309), DURAClone IM T cell subsets Tube (B53328; both from Beckman Coulter, Krefeld, Germany) or with the following liquid antibodies of the MoT test: CD45RA FITC (A07786), CD197 PE (B30632), CD279 PC5.5 (B36123), CD14 PC7 (A22331), CD4 APC (IM2468), CD8 AA700 (B49181), CD3 AA750 (A94680), CD16 PB (B36292) and CD45 KrO (B36294), all from Beckman Coulter, Krefeld, Germany and the following PE/Dazzle 594 conjugated liquid antibodies CD19 (#302252), CD20 (#302348), CD56 (#318348) and CD66b (#396912) from BioLegend, San Diego, CA. Data were recorded with a Navios™ cytometer running Cytometry List Mode Data Acquisition and Analysis Software version 1.3 (Beckman Coulter). Analyses were performed using Kaluza software version 2.1 (Beckman Coulter).

### Statistics and Visualisation

Data visualisation, significance tests, regression analyses and correlations were calculated using GraphPad Prism version 9.1.1 (GraphPad Software, Inc., La Jolla, USA).

### SPADE Analysis

For advanced visualisation and reduction of dimensionality, multi-parameter flow cytometry data of melanoma patients were analyzed using the SPADE algorithm available on Cytobank Premium platform (Cytobank Inc., Santa Clara, CA). Briefly, whole EDTA blood samples were processed using the MoT test. Acquired data were analyzed in Kaluza (software version 2.1) according to the described gating strategy. Compensated events were exported through the CD45^+^ Leucocyte gate and uploaded to Cytobank. After scaling and gating of uploaded data, we ran a SPADE analysis on Lin^-^ cells with a target number of nodes set to 200 and a density-dependent downsampling target of 10%. SPADE clusters were re-exported and further analyzed using Kaluza software version 2.1 (Beckman Coulter, Krefeld, Germany).

## Data Availability Statement

Relevant data are available from the corresponding author upon reasonable request.

## Ethics Statement

The studies involving human participants were reviewed and approved by Ethics Committee University of Regensburg. The patients provided their written informed consent to participate in this study.

## Author Contributions

H-LS, JAH, and KK designed experiments, analyzed data and wrote the manuscript. GG provided expert statistical advice. MK provided expert flow cytometry advice. NA provided expert opinion in Laboratory Medicine. PR, LC, and FB revised the manuscript. HS and EG provided infrastructural support. SH provided clinical samples and expert Dermatological opinion. All authors contributed to the article and approved the submitted version.

## Funding

The authors gratefully acknowledge the financial support of the Bristol-Myers Squibb (BMS) Immune Oncology Foundation (Award FA19-009).

## Conflict of Interest

MK is a Beckman Coulter Life Sciences associate. SH has received consulting fees and speaker’s honoraria from BMS and Merck Sharp & Dohme (MSD). NA is employed by MVZ for Laboratory Medicine Raubling.

The remaining authors declare that the research was conducted in the absence of any commercial or financial relationships that could be construed as a potential conflict of interest.

## Publisher’s Note

All claims expressed in this article are solely those of the authors and do not necessarily represent those of their affiliated organizations, or those of the publisher, the editors and the reviewers. Any product that may be evaluated in this article, or claim that may be made by its manufacturer, is not guaranteed or endorsed by the publisher.

## Abbreviations

AA700, Allophycocyanin Alexa Fluor^®^ 700 [Alexa Fluor^®^ 700 carboxylic acid, succinimidyl ester, tris(triethylammonium salt)] (A700); AA750, Allophycocyanin Alexa Fluor^®^ 750 [Alexa Fluor^®^ 750 carboxylic acid, succinimidyl ester, tris (triethylammonium salt)] (APC-A750); APC, Allophycocyanin; ASR, Analyte Specific Reagent; B.H., Benjamini-Hochberg procedure; BMS, Bristol-Myers Squibb; CE, Conformité Européenne; CMV, cytomegalovirus; CTCAE, Common Terminology Criteria for Adverse Events; CTLA-4, cytotoxic T-lymphocyte-associated protein 4; CV, coefficient of variation; EDTA, ethylenediaminetetraacetic acid; EU, European Union; FITC, Fluoresceinisothiocyanate; FDR, False Discovery Rate; h, hour; ICB, immune checkpoint blockade; ICI, immune checkpoint inhibitor; Ig, immunoglobulin; irAE, immune-related adverse events; IVDR, *in vitro* diagnostic regulation; KrO, Krome Orange; K.W. test, Kruskal Wallis test; Lin, lineage; MFI, mean fluorescence intensity; min, minute; NK cell, natural killer cell; NPV, negative predictive value; PB, Pacific Blue; PC5.5, Phycoerythrin-Cyanine 5.5; PC7, Phycoerythrin-Cyanine 7; PD-1, programmed cell death protein 1; PE, Phycoerythrin; PPV, positive predictive value; RT, room temperature; RUO, research use only; SI, stain index; SD, standard deviation; SPADE, spanning-tree progression analysis of density-nomalized events; T_CM_, central memory T cells; T_EM_, effector memory T cells; T_EMRA_, CD45RA^+^ effector memory T cells; T_naive_, naive T cells; UKR, University Hospital Regensburg.
