# Supplementary material for: Development of a Flow Cytometry Assay to Predict Immune Checkpoint Blockade-Related Complications
Source: Front Immunol. 2021 Nov 16;12:765644. doi: 10.3389/fimmu.2021.765644 (PMC8637156; doi:10.3389/fimmu.2021.765644)
Supplement: Supplementary file 1 [file Presentation_1.pdf]

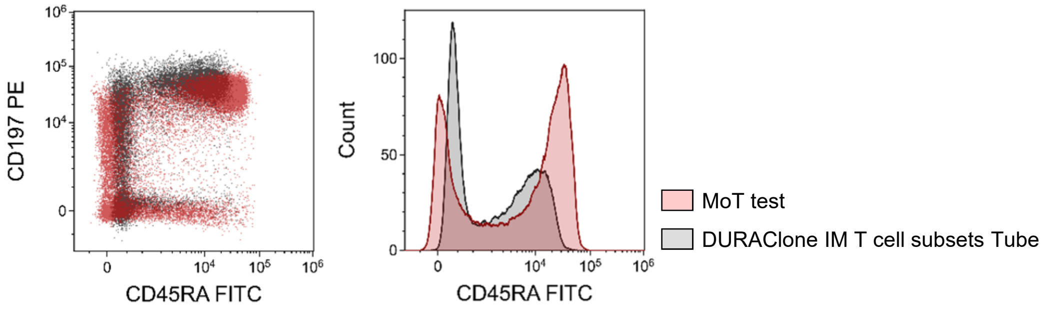


| | | $MFI_{neg}$ | $MFI_{pos}$ | $SD_{neg}$ | **Stain Index** |
|---|---|---|---|---|---|
| CD45RA FITC | MoT test | 102.67 | 15970.43 | 264.20 | **30.03** |
| | DURAClone IM T cell subsets Tube | 362.15 | 5544.72 | 176.97 | **14.64** |

**Supplementary Figure 1.** Two different clones of CD45RA FITC mAb are commercially available from Beckman Coulter. Clone *2H4* is included in the DURAClone IM T cell subsets Tube, clone *ALB11* was tested for the MoT test. To compare both clones, fresh whole EDTA blood from a healthy donor was stained in parallel with the MoT test (clone *ALB11*) and the DURAClone IM T cell subsets tube (clone *2H4*). A higher CD45RA FITC stain index was observed for clone *ALB11* (30.03 vs. 14.64).

| Parameter | CV (%) | | | | | | | | | SD | | | | | | | | |
|---|---|---|---|---|---|---|---|---|---|---|---|---|---|---|---|---|---|---|
| | | Sample | | | | | | | | | Sample | | | | | | | |
| | mean | 1 | 2 | 3 | 4 | 5 | 6 | 7 | 8 | mean | 1 | 2 | 3 | 4 | 5 | 6 | 7 | 8 |
| Leucocytes | 1,78 | 0,66 | 0,75 | 0,10 | 0,69 | 9,43 | 0,49 | 1,18 | 0,99 | 1,50 | 0,63 | 0,72 | 0,10 | 0,66 | 7,32 | 0,47 | 1,13 | 0,97 |
| $CD3^-$ | 1,84 | 0,63 | 2,03 | 0,99 | 3,27 | 1,92 | 2,54 | 1,46 | 1,85 | 1,30 | 0,55 | 1,49 | 0,80 | 2,13 | 1,40 | 1,65 | 1,02 | 1,32 |
| $CD3^+$ | 4,65 | 3,92 | 5,60 | 3,99 | 5,98 | 5,04 | 4,72 | 3,45 | 4,52 | 1,26 | 0,53 | 1,46 | 0,77 | 2,07 | 1,35 | 1,64 | 1,01 | 1,28 |
| $CD4^+$ | 0,65 | 0,54 | 0,92 | 0,43 | 0,63 | 0,66 | 0,50 | 1,03 | 0,49 | 0,37 | 0,19 | 0,56 | 0,29 | 0,34 | 0,40 | 0,31 | 0,57 | 0,33 |
| $CD8^+$ | 1,01 | 0,60 | 1,43 | 1,26 | 0,74 | 1,17 | 0,49 | 1,02 | 1,34 | 0,32 | 0,36 | 0,39 | 0,35 | 0,29 | 0,38 | 0,13 | 0,35 | 0,35 |
| $CD4^+$ $T_{EMRA}$ | 5,02 | 7,03 | 6,06 | 8,22 | 2,30 | 7,75 | 2,81 | 3,66 | 2,28 | 0,15 | 0,41 | 0,25 | 0,08 | 0,12 | 0,11 | 0,05 | 0,09 | 0,07 |
| $CD4^+$ $T_{CM}$ | 1,52 | 0,76 | 2,75 | 2,20 | 0,48 | 1,58 | 1,11 | 2,37 | 0,91 | 0,42 | 0,28 | 0,60 | 0,59 | 0,20 | 0,57 | 0,36 | 0,46 | 0,34 |
| $CD4^+$ $T_{EM}$ | 2,84 | 2,44 | 2,83 | 4,97 | 1,84 | 2,09 | 2,32 | 3,37 | 2,85 | 0,36 | 0,81 | 0,40 | 0,21 | 0,33 | 0,23 | 0,25 | 0,30 | 0,36 |
| $CD4^+$ $T_{NAIVE}$ | 1,23 | 3,91 | 0,57 | 1,10 | 0,98 | 0,66 | 0,86 | 0,79 | 0,98 | 0,53 | 0,95 | 0,34 | 0,75 | 0,34 | 0,34 | 0,48 | 0,55 | 0,46 |
| $CD279^+$ $CD8^+$ | 1,67 | 1,74 | 2,75 | 2,47 | 0,76 | 1,39 | 1,71 | 1,09 | 1,51 | 0,49 | 0,46 | 0,87 | 0,47 | 0,36 | 0,44 | 0,44 | 0,34 | 0,54 |
| $LIN^-$ | 5,97 | 2,92 | 4,30 | 4,93 | 5,58 | 8,33 | 6,96 | 9,68 | 5,03 | 0,60 | 0,32 | 0,55 | 0,32 | 0,79 | 0,71 | 0,58 | 0,92 | 0,58 |
| $CD14^+$ | 6,61 | 3,82 | 5,49 | 4,85 | 5,88 | 11,19 | 8,03 | 9,06 | 4,55 | 0,40 | 0,32 | 0,42 | 0,21 | 0,46 | 0,58 | 0,34 | 0,56 | 0,31 |
| $CD14^{high}$ $CD16^-$ | 4,42 | 3,34 | 7,95 | 1,89 | 5,13 | 5,95 | 5,94 | 3,28 | 1,90 | 2,42 | 2,23 | 3,10 | 1,56 | 3,35 | 2,25 | 3,18 | 2,30 | 1,37 |
| $CD14^{high}$ $CD16^+$ | 10,79 | 11,21 | 5,77 | 16,24 | 10,40 | 3,54 | 7,46 | 23,00 | 8,67 | 1,84 | 1,91 | 2,45 | 1,20 | 1,91 | 1,50 | 2,13 | 2,50 | 1,12 |
| $CD14^{low}$ $CD16^-$ | 7,30 | 7,85 | 6,80 | 7,70 | 7,77 | 5,77 | 8,63 | 9,36 | 4,51 | 0,24 | 0,24 | 0,22 | 0,29 | 0,21 | 0,27 | 0,33 | 0,21 | 0,14 |
| $CD14^{low}$ $CD16^+$ | 6,06 | 3,65 | 6,95 | 5,48 | 11,71 | 8,89 | 5,99 | 2,74 | 3,09 | 0,81 | 0,48 | 1,05 | 0,35 | 1,59 | 1,36 | 0,85 | 0,46 | 0,36 |

**Supplementary Figure 2.** For evaluation of intra-assay precision, fresh whole blood EDTA samples from eight healthy donors were stained in six replicates (n=48) using the MoT test. Standard Deviation (SD) and Coefficient of Variation (CV) were calculated for every parameter. Reporters' CV for intra-assay precision (marked in red) are all below 10 %.

| Parameter | CV (%) | | | | | | | | | SD | | | | | | | | |
|---|---|---|---|---|---|---|---|---|---|---|---|---|---|---|---|---|---|---|
| | | Sample | | | | | | | | | Sample | | | | | | | |
| | mean | 1 | 2 | 3 | 4 | 5 | 6 | 7 | 8 | mean | 1 | 2 | 3 | 4 | 5 | 6 | 7 | 8 |
| Leucocytes | 0,50 | 0,86 | 0,39 | 0,35 | 0,41 | 0,06 | 0,54 | 0,26 | 0,44 | 0,48 | 0,81 | 0,38 | 0,34 | 0,39 | 0,06 | 0,52 | 0,26 | 0,42 |
| $CD3^-$ | 1,44 | 1,49 | 0,70 | 0,75 | 2,81 | 0,38 | 0,51 | 0,43 | 0,64 | 1,04 | 1,09 | 0,54 | 0,56 | 1,99 | 0,31 | 0,32 | 0,36 | 0,58 |
| $CD3^+$ | 3,74 | 4,08 | 2,29 | 1,99 | 6,59 | 1,70 | 0,90 | 2,23 | 6,09 | 1,00 | 1,08 | 0,53 | 0,50 | 1,90 | 0,29 | 0,33 | 0,36 | 0,58 |
| $CD4^+$ | 0,82 | 2,23 | 0,53 | 0,42 | 0,12 | 0,32 | 0,32 | 0,38 | 0,55 | 0,45 | 1,19 | 0,26 | 0,25 | 0,07 | 0,19 | 0,19 | 0,26 | 0,37 |
| $CD8^+$ | 1,29 | 3,06 | 0,89 | 0,86 | 0,33 | 1,05 | 0,77 | 0,93 | 1,32 | 0,48 | 1,17 | 0,35 | 0,29 | 0,10 | 0,30 | 0,24 | 0,21 | 0,36 |
| $CD4^+$ $T_{EMRA}$ | 6,14 | 5,44 | 8,35 | 4,39 | 6,38 | 6,83 | 1,79 | 2,29 | 0,87 | 0,08 | 0,12 | 0,10 | 0,06 | 0,04 | 0,07 | 0,12 | 0,04 | 0,03 |
| $CD4^+$ $T_{CM}$ | 0,68 | 0,73 | 0,78 | 0,63 | 0,55 | 1,74 | 1,71 | 1,15 | 2,37 | 0,20 | 0,23 | 0,21 | 0,24 | 0,13 | 0,46 | 0,57 | 0,21 | 0,70 |
| $CD4^+$ $T_{EM}$ | 4,55 | 5,88 | 6,15 | 4,37 | 1,80 | 7,11 | 4,57 | 7,35 | 7,38 | 0,41 | 0,56 | 0,48 | 0,45 | 0,14 | 0,28 | 0,58 | 0,28 | 1,23 |
| $CD4^+$ $T_{NAIVE}$ | 0,67 | 0,83 | 0,30 | 1,18 | 0,36 | 0,46 | 0,59 | 0,70 | 2,29 | 0,37 | 0,48 | 0,19 | 0,58 | 0,25 | 0,31 | 0,28 | 0,53 | 1,15 |
| $CD279^+$ $CD8^+$ | 2,36 | 0,26 | 1,90 | 6,10 | 1,20 | 1,32 | 0,78 | 4,43 | 2,18 | 1,03 | 0,13 | 0,57 | 2,88 | 0,52 | 0,25 | 0,36 | 0,75 | 1,16 |
| $LIN^-$ | 6,72 | 3,05 | 6,95 | 6,36 | 10,52 | 1,83 | 13,31 | 5,78 | 6,95 | 0,73 | 0,39 | 0,57 | 0,70 | 1,26 | 0,14 | 1,75 | 0,71 | 1,18 |
| $CD14^+$ | 7,81 | 1,22 | 7,37 | 7,45 | 15,25 | 1,58 | 14,24 | 7,42 | 7,95 | 0,57 | 0,10 | 0,40 | 0,52 | 0,90 | 0,09 | 0,87 | 0,61 | 1,08 |
| $CD14^{high}$ $CD16^-$ | 9,64 | 1,19 | 3,50 | 6,73 | 27,15 | 0,17 | 12,99 | 11,10 | 13,45 | 4,64 | 0,79 | 2,58 | 4,57 | 10,62 | 0,15 | 5,60 | 3,84 | 5,81 |
| $CD14^{high}$ $CD16^+$ | 18,12 | 8,10 | 10,70 | 32,82 | 20,85 | 4,03 | 14,21 | 6,14 | 12,62 | 3,46 | 0,69 | 1,98 | 2,62 | 8,53 | 0,16 | 3,89 | 2,58 | 4,84 |
| $CD14^{low}$ $CD16^-$ | 8,85 | 9,19 | 7,30 | 12,04 | 6,89 | 3,27 | 9,93 | 5,86 | 3,89 | 0,25 | 0,19 | 0,18 | 0,41 | 0,23 | 0,10 | 0,31 | 0,10 | 0,09 |
| $CD14^{low}$ $CD16^+$ | 8,23 | 1,87 | 7,61 | 7,91 | 15,51 | 2,16 | 7,60 | 5,38 | 7,00 | 1,27 | 0,44 | 0,42 | 1,64 | 2,58 | 0,07 | 2,01 | 1,17 | 1,14 |

**Supplementary Figure 3.** For evaluation of inter-assay precision, fresh whole EDTA blood from eight healthy donors was stained in parallel by three different operators (n=24) using the MoT test. Standard Deviation (SD) and Coefficient of Variation (CV) were calculated for every parameter. Reporters' CV for inter-assay precision (marked in red) are all below 10 %.

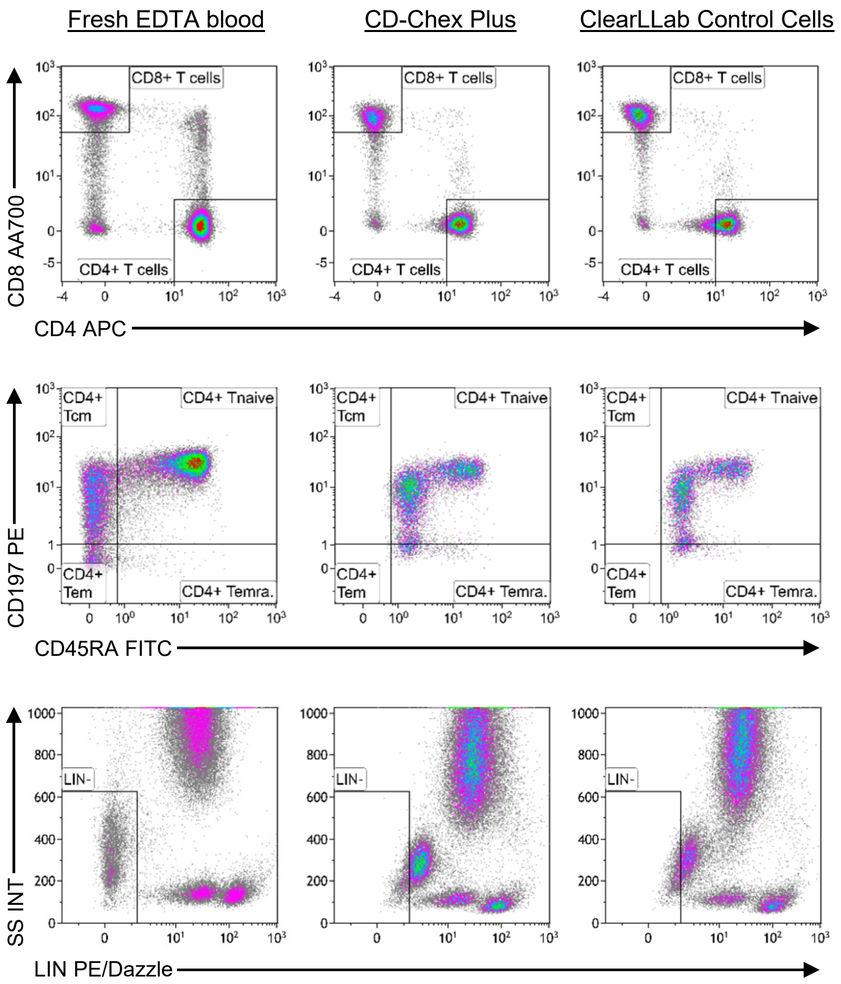


**Supplementary Figure 4.** Commercially available reference material could be used to continuously confirm the assays performance. Here, we compared fresh EDTA blood with Streck CD-Chex Plus and Beckman Coulter ClearLLab Control Cells Normal. Samples were stained in parallel using the MoT test. The external reference material was not comparable to cells from fresh blood. In particular, fluorescence intensities of CD4 APC, CD45RA FITC and LIN PE/Dazzle clearly differ from EDTA blood. However, the manufacturers' provided reference quality control data could be reproduced by the MoT test. Based on these acceptance criteria, the measurements' accuracy could be verified over time.

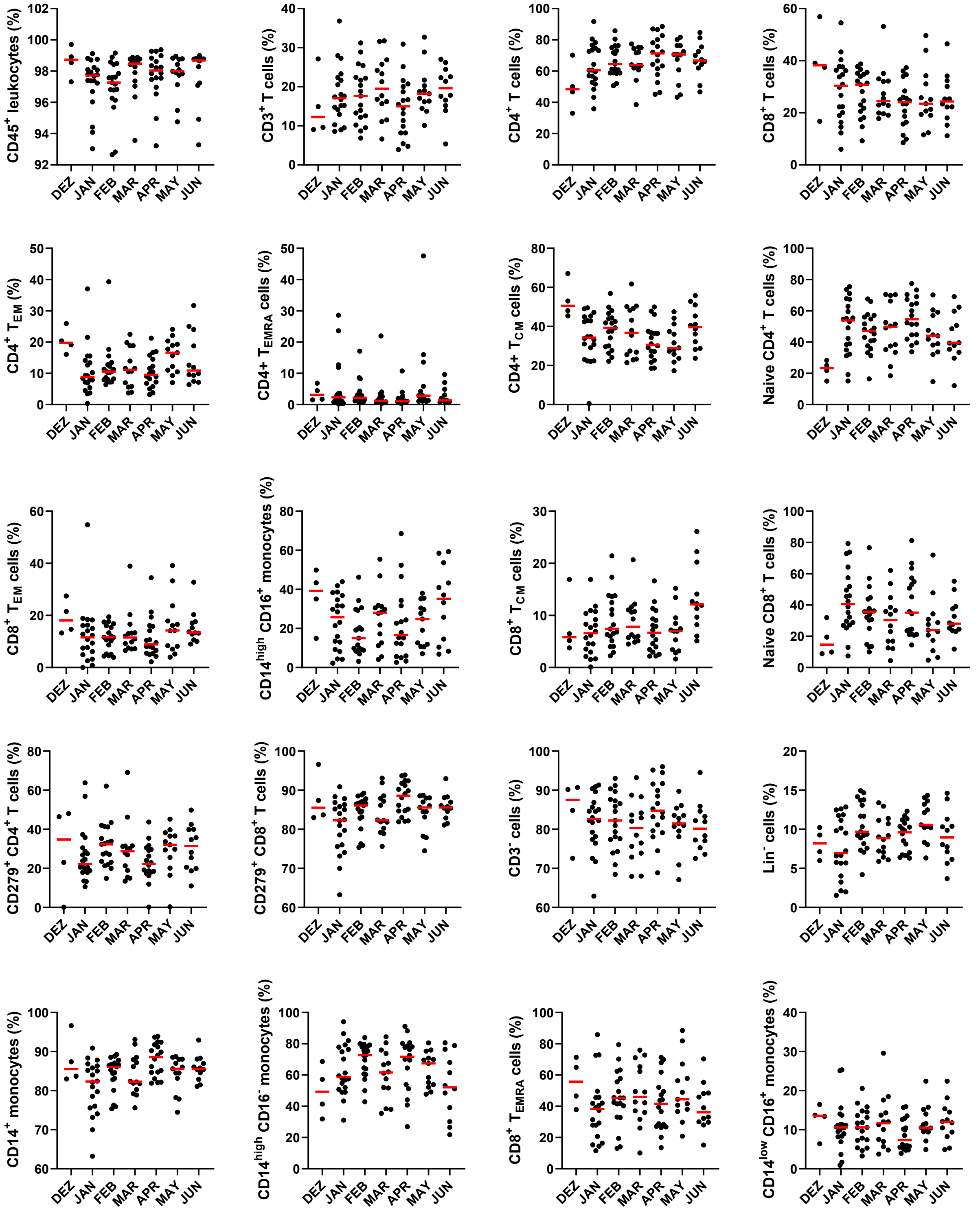


**Supplementary Figure 5.** Fresh whole EDTA blood from 100 melanoma patients (stage II: n=52; stage III: n=21; stage IV: n=27) was stained using the MoT test. Flow cytometry data were analyzed by Kaluza (software version 2.1) using the described gating strategy (Figure 2). Seasonality for every parameter analyzed is shown.

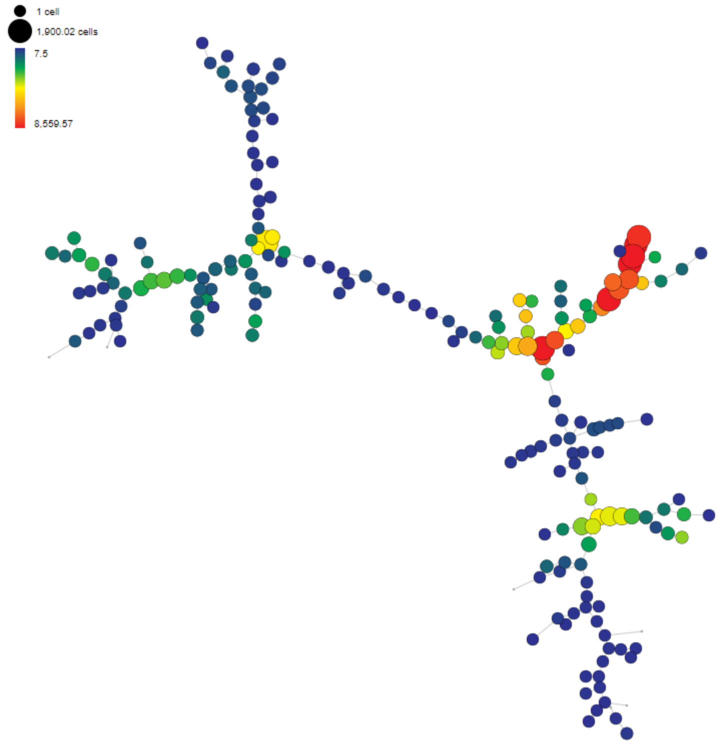


**Supplementary Figure 6.** Fresh whole EDTA blood from 100 melanoma patients (stage II: n=52; stage III: n=21; stage IV: n=27) was stained using the MoT test. Flow cytometry data were uploaded into Cytobank and a SPADE analysis of Lin$^{-}$ cells was done with a density-dependent downsampling target of 10 % and a 200 nodes spanning tree. One Stage II SPADE tree is shown as an example. Nodes are colored by density.

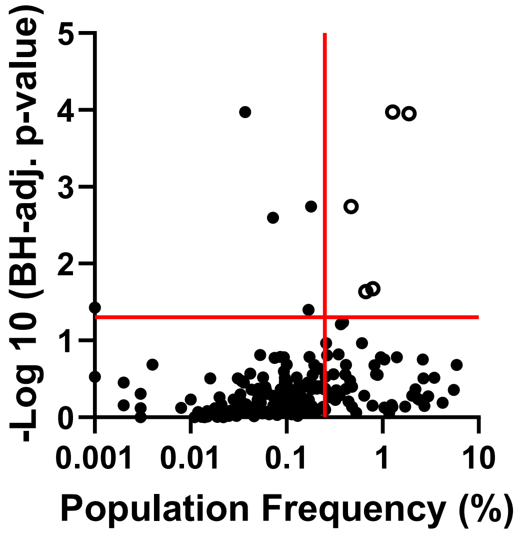


**Supplementary Figure 7.** Node frequencies of the SPADE analysis were analyzed using a p-value cut-off of α=0.5 and a frequency cut-off of 0.25 % (red lines). Five statistically significant nodes were identified (circles).

| Standard Protocol for the MoT test | | |
|---|---|---|
| **Step** | **Action** | **Check** |
| 1 | Collect peripheral blood samples into EDTA-vacutainers by venepuncture | ☐ |
| 2 | Pre-analytical samples can be stored up to 4 hrs at 4°C until processing | ☐ |
| 3 | Pipet antibodies into a FACS tube according to table | ☐ |
| 4 | Mix the blood sample by carefully inverting the vacutainer | ☐ |
| 5 | Transfer 100 µl blood into the FACS tube | ☐ |
| 6 | Vortex for 8 sec | ☐ |
| 7 | Incubate at room temperature in the dark for 15 min | ☐ |
| 8 | Add 2 ml VersaLyse | ☐ |
| 9 | Vortex for 3 sec | ☐ |
| 10 | Incubate at room temperature in the dark for 15 min | ☐ |
| 11 | Pellet cells by centrifugation at 200 g for 5 min | ☐ |
| 12 | Aspirate supernatant using vacuum pump | ☐ |
| 13 | Vortex for 3 sec | ☐ |
| 14 | Add 3 ml DPBS | ☐ |
| 15 | Pellet cells by centrifugation at 200 g for 5 min | ☐ |
| 16 | Aspirate supernatant using vacuum pump | ☐ |
| 17 | Resuspend cells in 380µl 1,25%-PBS-Fixative-Solution | ☐ |
| 18 | Cells are now ready for acquistion | ☐ |

| | **Reagents** | **Check** |
|---|---|---|
| 1 | Antibodies according to table (see Figure 1A) | ☐ |
| 2 | VersaLyse Solution (A09777, Beckman Coulter) | ☐ |
| 3 | DPBS without $Ca^{2+}$ or $Mg^{2+}$ (D8537, Sigma) | ☐ |
| 4 | IOTest 3 Fixative Solution (10x) (A07800, Beckman Coulter) | ☐ |

| | **Material** | **Check** |
|---|---|---|
| 1 | S-Monovette® 2,7 ml, K3 EDTA (05.1167, Sarstedt) | ☐ |
| 2 | Test Tube, 12x75 mm, blue (2523749, Beckman Coulter) | ☐ |

**Supplementary Method.** Standard Protocol for the MoT test.
